# Slot-Loaded Vivaldi Antenna for Biomedical Microwave Imaging Applications: Influence of Design Parameters on Antenna’s Dimensions and Performances

**DOI:** 10.3390/s24165368

**Published:** 2024-08-20

**Authors:** Mengchu Wang, Lorenzo Crocco, Maokun Li, Marta Cavagnaro

**Affiliations:** 1 Department of Electronic Engineering, Tsinghua University, Beijing 100084, China; maokunli@tsinghua.edu.cn; 2 CNR-IREA National Research Council of Italy, Institute for Electromagnetic Sensing of the Environment, 80124 Naples, Italy; crocco.l@irea.cnr.it; 3 Department of Information Engineering, Electronics, and Telecommunications, University of Rome “La Sapienza”, 00184 Rome, Italy; marta.cavagnaro@uniroma1.it

**Keywords:** Vivaldi antenna, biomedical microwave imaging, antenna miniaturization, coupling medium, suppress mutual coupling

## Abstract

This paper demonstrates the design steps of a slot-loaded Vivaldi antenna for biomedical microwave imaging applications, showing the influence of the design parameters on the antenna’s dimensions and performances. Several antenna miniaturization techniques were taken into consideration during the design: reduction in the electromagnetic wavelength by using a high-permittivity substrate material (relative permittivity $\epsilon_{r}=10.2$), the placement of the antenna inside a coupling medium ($\epsilon_{r}=23$), and the elongation of the current path by etching slots on each side of the radiator to reduce the antenna’s lowest resonant frequency without increasing its physical dimensions. Moreover, an analysis of different antenna slot design scenarios was performed considering different slot lengths, inclination angles, positions, and numbers. Considering the frequency range of microwave imaging (i.e., about 500 MHz–5 GHz) and the array arrangement typical of microwave imaging, the best design was chosen. Finally, the antenna was fabricated and its performances in the coupling medium were characterized. The simulation and measurement results showed good agreement between each other. In comparison with literature antennas, the one developed in this work shows wide bandwidth and compact dimensions.

## 1. Introduction

In recent decades, microwave imaging (MWI) has been extensively researched, finding applications across a wide range of technical fields, including ground penetrating radar [1], through-wall detection [2], remote sensing [3], and biomedical imaging [4]. Among these applications, microwave imaging’s utility in biomedical applications stands out due to its favored performance compared to other imaging modalities like X-ray, computed tomography (CT), magnetic resonance imaging (MRI), etc. [5]. In particular, MWI systems are characterized by the use of non-ionizing radiation, are cost-effective, portable, and capable of real-time imaging. At the core of the MWI system lies the critical role of the used antennas, which determine the extent of electromagnetic wave penetration into biological tissues [6]. Additionally, employing multiple antennas in an array configuration proves to be advantageous, enhancing available data and consequently refining imaging resolution [7]. This multi-static arrangement accelerates measurement speed by avoiding the mechanical movement of a mono-static configuration.

Compact sensors are essential in biomedical imaging systems due to spatial constraints within the detection area. Notably, electromagnetic waves experience significant attenuation as they penetrate into biological tissues with increasing frequency [8]. Consequently, the design of compact antennas becomes crucial to maintain good performances at low frequencies.

Vivaldi antenna is a promising candidate for biomedical applications due to its end-fire radiation pattern, its compact aperture dimension (thickness × width), and its capability of being used within an array configuration [9]. The wide bandwidth of the Vivaldi antenna provides more freedom when it comes to multi-frequency system design. However, the lowest frequency of operation is directly related to the Vivaldi antenna dimension (length × width), so miniaturization techniques should be applied to allow operations at lower frequencies without increasing the overall size. Scaling down the antenna’s physical size without degradation of its performance is a challenge that researchers have confronted over the years. The relation between the antenna’s physical dimension and quality factor Q was early discussed by Wheeler [10], Chu [11] and Hansen [12]: the shrink of the antenna’s physical size always comes together with the reduction in its bandwidth and Q factor.

A first technique for antenna miniaturization is to fit the limited space with larger radiating structures [13], using, e.g., meander-line design [14] or fractal structures [15]. It is well known that increasing the surface current path of an antenna is an efficient way to reduce antenna size [16]. Promising studies such as [17,18,19,20,21] demonstrated the possibility of reducing the Vivaldi antenna’s physical dimensions by using either circular or corrugated slots on the antenna radiator. A second technique is to use metamaterials [22] as, e.g., split-resonant-ring (SRR), and add resonances to the antenna. The third technique is to incorporate reactive loads into the antenna transmission line structure [13]. This introduces time delay and slows down the electromagnetic wave propagation, hence making the transmission line electrically longer. However, due to the limited Q factor of the reactive load, the antenna benefit of dimension reduction always comes with efficiency losses [23].

The goal of this study was to show the design approach of a miniaturized Vivaldi antenna for biomedical applications. The concerned frequency range was between 500 MHz and 5 GHz, typical of MWI systems [24,25]. To show the influence of the design parameters on the antenna’s dimensions and performances, a class of antenna miniaturization techniques was investigated. Firstly, the reduction in the electromagnetic wavelength was explored, which was achieved by employing an antenna substrate material with a high relative permittivity ($\epsilon_{r}=10.2$) and situating the antenna within a coupling medium ($\epsilon_{r}=23$). The results in [26] showed that using a high-permittivity substrate and a high-permittivity coupling medium efficiently reduced the antenna’s width to 0.4λ. However, it proved challenging to further reduce the antenna’s physical size by modifying the antenna structure. Accordingly, in this study, further reduction in the antenna’s dimensions was looked for by extending the current path of the antenna etching slots on both sides of the radiator. Different antenna slot configurations were studied. In particular, the influence of different slot lengths, positions, inclination angles, and numbers of slots on the antenna bandwidth were studied. Among the study designs, a four-slot Vivaldi antenna was considered the optimal choice for MWI applications. The proposed antenna was fabricated, and its performances were experimentally characterized inside the coupling medium and in an array configuration.

The paper’s structure is as follows: Section 2 presents the study methodology. In Section 3, the basic antenna structure is given. In Section 4, a range of slot configurations are discussed, focusing on their potential to enhance antenna matching at lower frequencies. This section also investigates the mutual coupling effect of the antennas when arranged in an array configuration. Moreover, it examines the E-field distribution of the antenna and its performance in the presence of a phantom. The comparison of the antenna’s simulation and measurement results are reported in this section as well. Finally, Section 5 presents the paper’s conclusion.

## 2. Methodology

**Figure 1:**
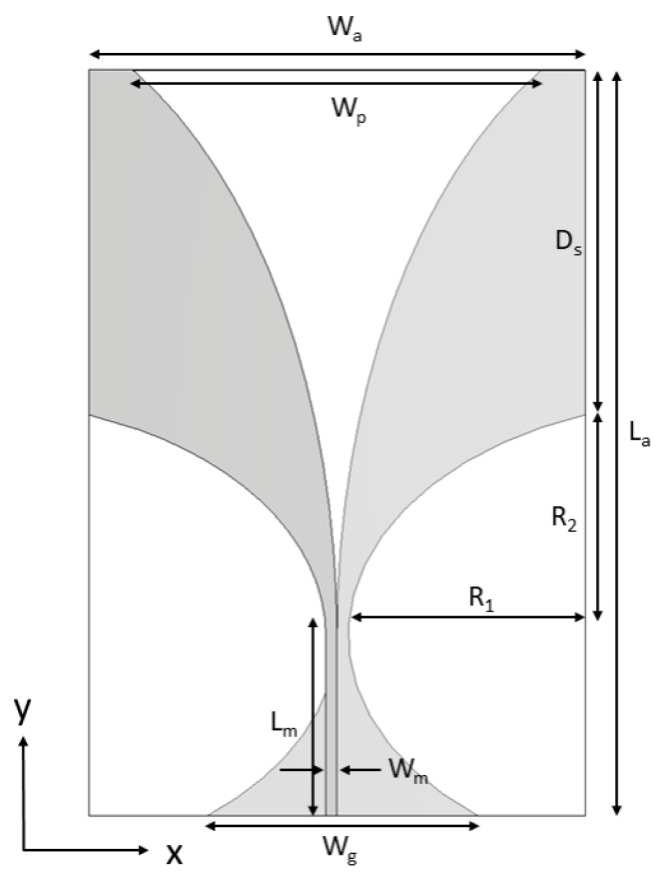
Geometry of the basic antipodal Vivaldi antenna.

The antenna was designed and optimized with CST Studio Suite R2021x software (Dassault Systèmes SE, Vélizy-Villacoublay, France). The basic antenna geometry shown in Figure 1 was first optimized by using the parameter sweep function on the following parameters: $W_{p}$, $W_{g}$, $W_{m}$, $D_{s}$, $L_{a}$, $L_{m}$, $R_{1}$, $R_{2}$, looking for the widest bandwidth. Then, the insertion of slots to allow reducing antenna’s dimensions while keeping its matching in the desired frequency band was considered. Since the interest was in investigating the influence of slot parameters on the antenna performances, antenna optimization was performed as a mix of physical considerations and software sweep analysis.

To study the impact of antenna slot configurations on the antenna impedance, the following slot configurations were investigated: one-slot, two-slot, three-slot, and four-slot configurations. In particular, studies on different slots lengths and inclination angles were performed.

Ultimately, the best-performing antenna was fabricated. It was realized by using a single-layered substrate TF1020 (TaiZhou WangLing, Taizhou, China. Thickness ts=1.905 mm, $\epsilon_{r}=10.2$). The experimental set-up for the antenna measurement consisted of an antenna connected with a coaxial cable and immersed inside a tank (dimensions: 200 mm × 200 mm × 200 mm) filled with the coupling medium. The coupling medium ($\epsilon_{r}=22.9$, σ=0.13 S/m @ 915 MHz) was made by following the recipe from [26]; it is a time-stable low-loss liquid medium consisting of distilled water, sunflower oil, guar gum, and dishwashing detergent. The measurement of the antenna scattering parameters inside the coupling medium was made by using the P5024A Vector Network Analyzer (Keysight Technologies, Santa Rosa, CA, USA).

## 3. Antenna Element Design

### Geometry of the Basic Antipodal Vivaldi Antenna Element

The geometry of the basic antipodal Vivaldi antenna is reported in Figure 1 [26]. Its dimensions are calculated through the following equation (modified from [24]):

(1) $$W_{a}=L_{a}=\frac{c}{f_{1}}\sqrt{\frac{2}{\epsilon_{mm}+\epsilon_{sub}}},$$

where $W_{a}$ is the antenna width, $L_{a}$ is the length, *c* is the speed of light in vacuum, $f_{1}$ is the lowest working frequency, $\epsilon_{mm}$ is the permittivity of the coupling medium, $\epsilon_{sub}$ is the permittivity of the substrate. Applying Equation (1), an antenna working from 500 MHz to 5 GHz in the proposed coupling medium and with dimensions of 60 mm × 60 mm is obtained (untrimmed antipodal Vivaldi antenna). To reduce the antenna width, the antenna was trimmed by 10 mm on each side, leading to an overall dimension of 40 mm × 60 mm. Table 1 reports the dimensions of the trimmed antenna.

## 4. Results

### 4.1. Basic Antipodal Vivaldi Antenna

**Figure 2:**
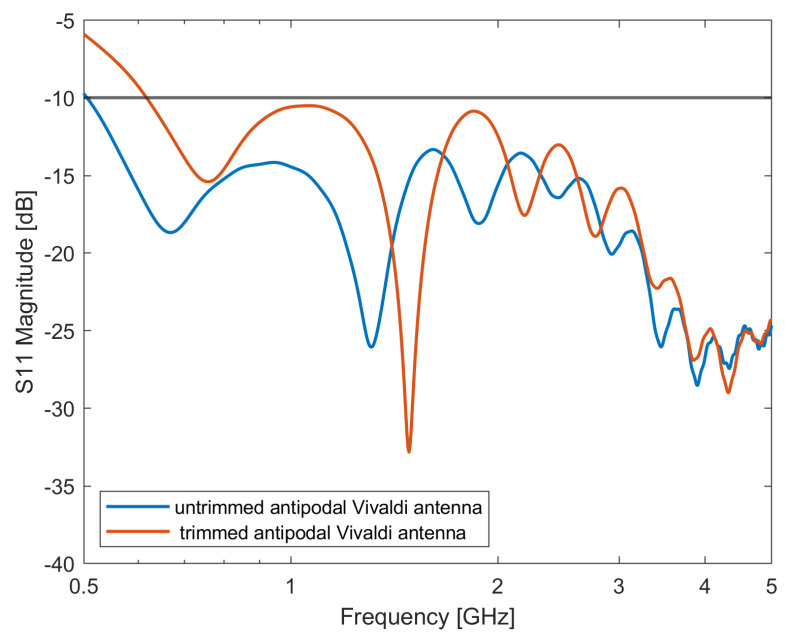
Simulated S-parameters of the basic antipodal Vivaldi antenna, bandwidth: 0.62–5 GHz.

Figure 2 reports the reflection coefficient of the untrimmed antipodal Vivaldi antenna and the antipodal Vivaldi antenna after the trim (basic antipodal Vivaldi antenna in Figure 1). Trimming the antenna is significant in reducing its physical size while maintaining its radiation performance at the higher frequencies. From Figure 2, it can be noted that the shrink of the antenna’s physical dimension inevitably shifts the antenna’s lowest working frequency from 500 MHz to 620 MHz. To reduce the antenna’s lowest working frequency, in [26], a single-slot-loaded configuration was proposed to elongate the current path on the radiator without increasing the antenna’s overall dimension. This study investigates the influence of different slot-loaded designs on antenna performances. Particularly, different slot lengths, positions, inclination angles, and slot numbers were studied. In the following, the antenna’s performances are reported up to 5 GHz, although the antenna is capable of operating at even higher frequencies. However, frequencies higher than 5 GHz are not used in microwave imaging applications mainly because of the poor penetration depth into the tissues. Due to the practical importance of lower frequencies for deeper tissue penetration in biomedical imaging, the study mainly focuses on frequencies up to 1.2 GHz.

### 4.2. Single-Slot-Loaded Antipodal Vivaldi Antenna Study

To start with, a single slot was placed in the middle of the radiator. The initial guess of the single-slot-loaded antenna design parameters is reported in Table 2, where it can be noted that the length of the antenna slot is the same as the one in [26] corresponding to a quarter wavelength in the coupling medium at 625 MHz.

#### 4.2.1. Variation of Slot Inclination Angle θ

**Figure 3:**
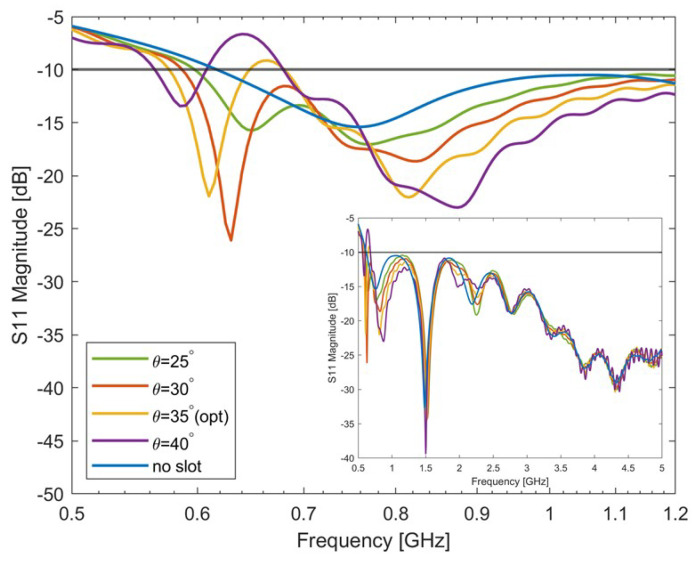
Simulated S-parameters of the Single slot-loaded antipodal Vivaldi antenna with different slot inclination angles. The optimal antenna’s bandwidth is: 580 MHz–645 MHz, 680 MHz–5 GHz. The antenna’s matching across the entire specified frequency range is reported in the figure insert.

The study on the variation of the slot inclination angle θ considered values from $25^{\circ }$ to $40^{\circ }$ in steps of $5^{\circ }$. The antenna S-parameters are reported in Figure 3. Indeed, as the antenna’s basic geometry remains, it is found that the variation of the slot configuration does not change the antenna bandwidth at higher frequencies (above 1.2 GHz). Therefore, in the figure, the S-parameter behaviour between 500 MHz and 1.2 GHz is reported, while the whole band is shown in the figure inset. In Figure 3, it can be seen that the antenna impedance changes as the inclination varies. In particular, the lowest frequency decreases for increasing angles but the matching worsens in a frequency band between about 620 MHz and 680 MHz. The antenna obtained for $\theta =35^{\circ }$, $L_{s}$ = 25 mm was considered the optimal choice in terms of the antenna working frequency, and it was chosen as the reference for the successive step.

#### 4.2.2. Variation of Slot Length $L_{s}$

**Figure 4:**
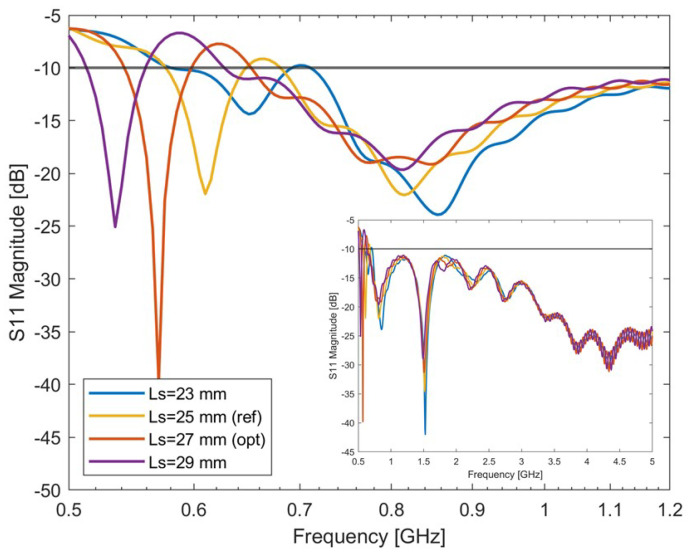
Simulated S-parameters of the single-slot-loaded antipodal Vivaldi antenna with different slot lengths $L_{s}$. The optimal antenna’s bandwidth is: 545 MHz–595 MHz, 655 MHz–5 GHz.

To further optimize the antenna performances, the influence of different antenna slot lengths $L_{s}$ was investigated. The design parameters are reported in Table 3, where it can be noted that the slot length was varied between 23 and 29 mm in steps of 2 mm. The antenna S-parameters are reported in Figure 4. It can be seen from the figure that the variation in slot length changes the antenna impedance and the antenna’s resonance shifts to a lower working frequency as the slot length extends. However, here, again, a mismatch is present between about 560 MHz and 620 MHz. $\theta =35^{\circ }$, ($L_{s}$ = 27 mm) was considered the optimal choice in terms of resonance and working frequency, and it was chosen as the reference for the next step.

#### 4.2.3. Variation of Slot Height $D_{1}$

**Figure 5:**
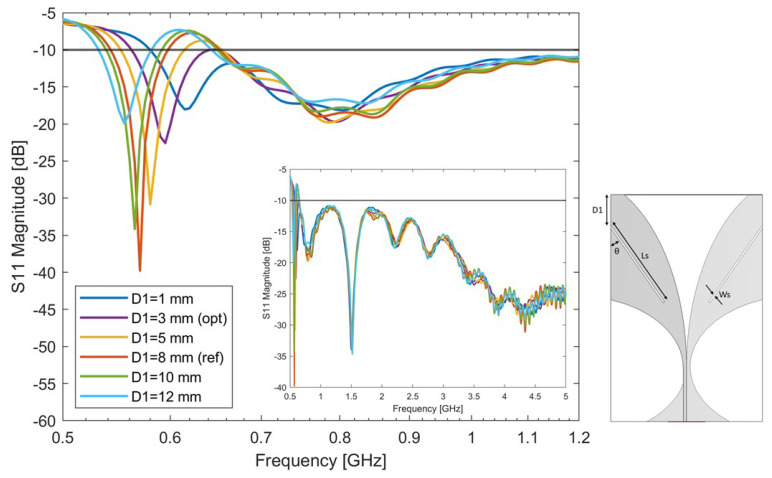
Simulated S-parameters of the single slot-loaded antipodal Vivaldi antenna with different $D_{1}$ values. The optimal antenna’s bandwidth is 565 MHz–5 GHz. The antenna’s matching across the entire specified frequency range is reported in the figure insert.

To further optimize the antenna performance, the influence of different slot heights $D_{1}$ was investigated. The design parameters are reported in Table 4, where it is shown that $D_{1}$ was varied between 1 and 12 mm. The antenna S-parameters are reported in Figure 5. It can be seen from the figure that the variation of the slot height changes the antenna impedance; in particular, the antenna’s resonance shifts to a lower working frequency as the slot height increases. At the same time, lower slot height allows for improvement in the matching in the whole considered frequency band. The optimal choice of $\theta =35^{\circ }$, $L_{s}$ = 27 mm, $D_{1}$ = 3 mm was considered in terms of bandwidth, and it was chosen as the reference for the next step.

### 4.3. Two-Slot-Loaded Antipodal Vivaldi Antenna Study

**Figure 6:**
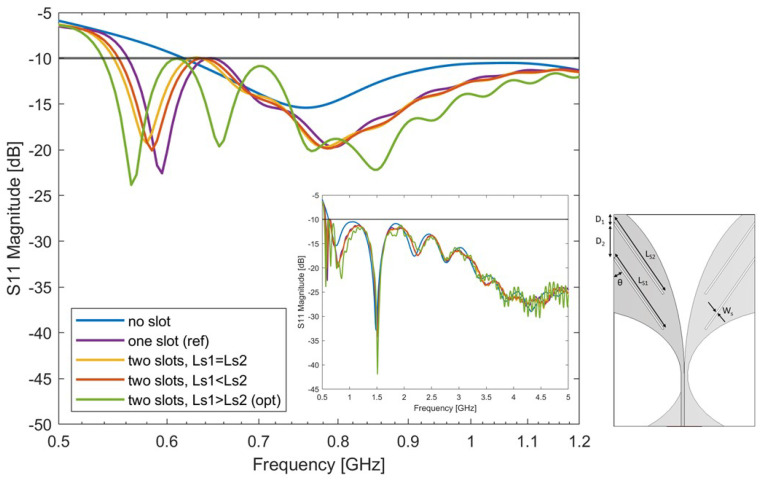
Simulated S-parameters of the two-slot-loaded antipodal Vivaldi antenna with different slot lengths $L_{s1}$ and $L_{s2}$. The optimal antenna’s bandwidth is: 540 MHz–5 GHz. The antenna’s matching across the entire specified frequency range is reported in the figure insert.

As it is known from the previous study, the variation of parameters $D_{1}$ and $L_{s}$ lead to a change in the antenna impedance. The addition of a slot to the basic Vivaldi design allowed reduction in the minimum working frequency of the antenna. However, this minimum frequency was still well above 500 MHz. In this section, two slots are etched on each side of the antenna’s radiator (see sub-plot of Figure 6). The antenna’s slot width and the inclination angle remain the same as in the previous section since the single-slot design with $\theta =35^{\circ }$, $L_{s}$ = 27 mm, $D_{1}=3$ mm is the reference design from the previous step. Three different scenarios are discussed, i.e., $L_{s1}$ = $L_{s2}$, $L_{s1}$ > $L_{s2}$, and $L_{s1}$ < $L_{s2}$. Antenna parameters of each scenario were selected through an optimization process looking for the lowest working frequency and the widest bandwidth. The following parameters were taken into consideration during the optimization: $D_{1}$, $D_{2}$, $L_{s1}$, and $L_{s2}$.

The optimal results looking for the lowest working frequency and widest bandwidth of each scenario are reported in Figure 6. The corresponding parameters are reported in Table 5. It can be seen from the figure that when $L_{s1}$ = $L_{s2}$, and $L_{s1}$ < $L_{s2}$, the antenna’s performance is similar to that of the single-slot antenna. When length $L_{s1}$ is larger than length $L_{s2}$, the antenna has the lowest resonance frequency. The slot lengths $L_{s1}$ and $L_{s2}$ correspond to a quarter wavelength in the medium at 560 MHz and 625 MHz, close to where the first and second resonances of the antenna show up.

### 4.4. Three-Slot-Loaded Antipodal Vivaldi Antenna Study

**Figure 7:**
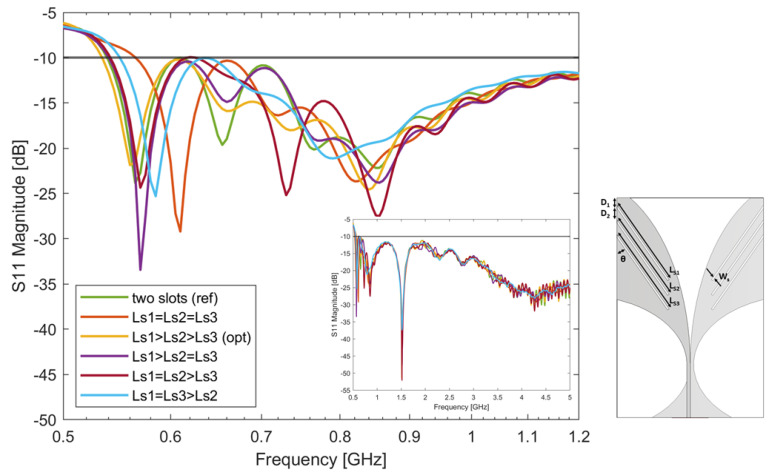
Simulated S-parameters of the three-slot-loaded antipodal Vivaldi antenna with different slot lengths $L_{s1}$, $L_{s2}$, and $L_{s3}$. The optimal antenna’s bandwidth is: 535 MHz–5 GHz. The antenna’s matching across the entire specified frequency range is reported in the figure insert.

Similar to the two-slot study, a three-slot configuration was studied. In this study, three slots were etched on each side of the antenna’s radiator (see inset in Figure 7). The following different scenarios were considered: $L_{s1}$ = $L_{s2}$ = $L_{s3}$, $L_{s1}$ > $L_{s2}$ > $L_{s3}$, $L_{s1}$ > $L_{s2}$ = $L_{s3}$, $L_{s1}$ = $L_{s2}$ > $L_{s3}$, and $L_{s1}$ = $L_{s3}$ > $L_{s2}$. The optimal results looking for the lowest working frequency and widest bandwidth of each scenario are reported in Figure 7. The corresponding parameters are reported in Table 6. It was found that when $L_{s1}$ > $L_{s2}$ > $L_{s3}$ the antenna has the lowest working frequency and widest bandwidth. The slot lengths $L_{s1}$, $L_{s2}$, and $L_{s3}$ correspond to a quarter wavelength in the medium at 560 MHz, 600 MHz, and 650 MHz. It is interesting to note that resonances are present at about 560 MHz and above 650 MHz, showing that the three slots interact one with the others in the determination of the antenna impedance. As the length differences between $L_{s1}$, $L_{s2}$, and $L_{s3}$ are not significant, the second and the third resonance of the antenna are less evident compared to the two-slot scenario. In addition, the scenarios of $L_{s1}$ > $L_{s2}$ = $L_{s3}$ and $L_{s1}$ = $L_{s2}$ > $L_{s3}$ have the same bandwidth because the longest slot $L_{s1}$ is the same in both scenarios.

It is worth noticing here that the change in the lower frequency achieved with the three-slot antenna, with respect that of the two-slot one, is of about 5 MHz only; however, the wideband behaviour of the $S_{11}$ is generally improved. In particular, the optimal three-slot scenario demonstrates an improvement of matching at around 700 MHz with respect the reference two-slot scenario.

### 4.5. Four-Slot-Loaded Antipodal Vivaldi Antenna Study (Final Design)

**Figure 8:**
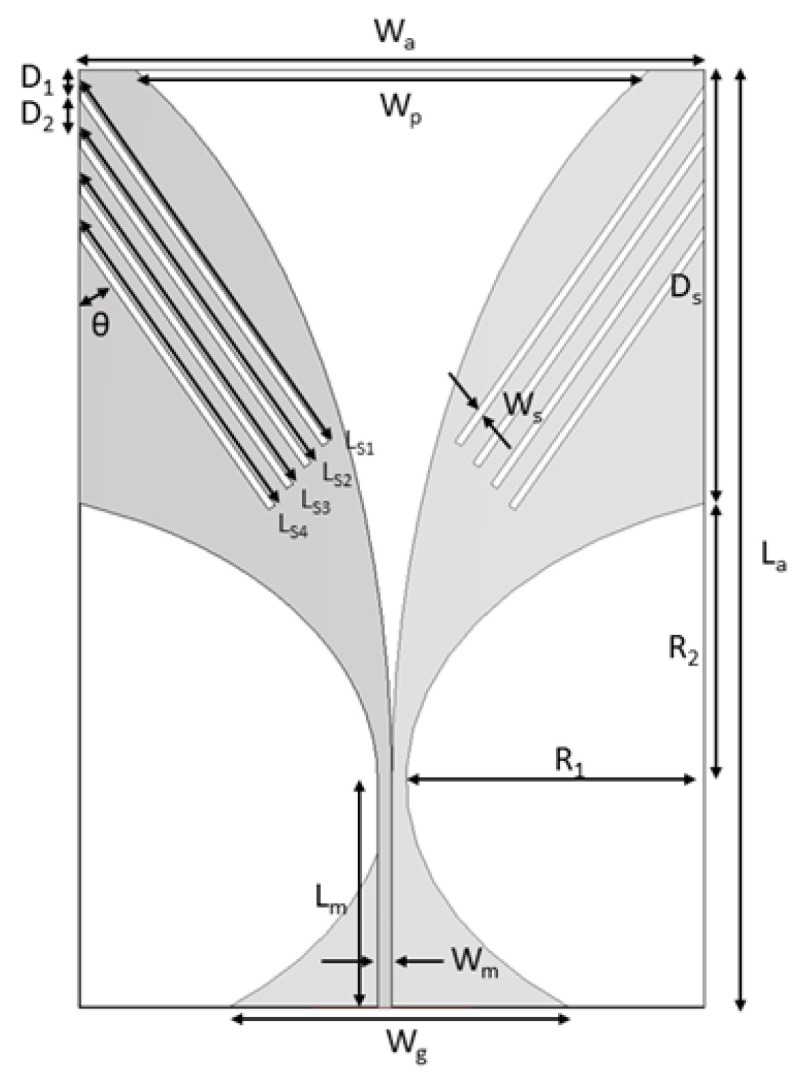
Geometry of the four-slot-loaded antipodal Vivaldi antenna element (Final design).

**Figure 9:**
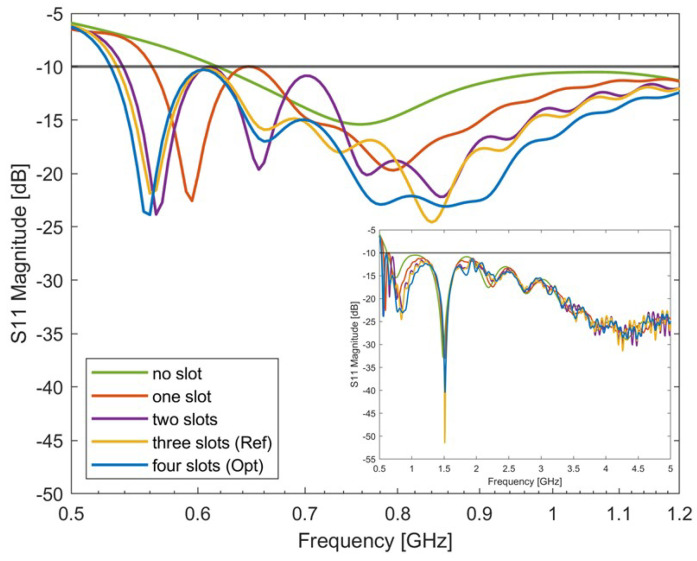
Simulated S-parameters of the slot-loaded antipodal Vivaldi antennas with different slot numbers. The optimal antenna’s bandwidth is: 530 MHz–5 GHz. The antenna’s matching across the entire specified frequency range is reported in the figure insert.

Similar to the three-slot study, four-slot-loaded configurations were studied. In this study, four slots were etched on each side of the antenna’s radiator (Figure 8). In particular, the following scenario was considered: $L_{s1}$ > $L_{s2}$ > $L_{s3}$ > $L_{s4}$. The optimal results looking for the lowest working frequency and widest bandwidth are reported in Figure 9 compared with the other antennas with one, two, or three slots. The parameters for the four-slot scenario are reported in Table 7. The slot lengths $L_{s1}$, $L_{s2}$, $L_{s3}$, and $L_{s4}$ correspond to a quarter wavelength in the medium at 560 MHz, 600 MHz, 650 MHz, and 710 MHz. Due to the interaction of the slots, the resonances of the antenna show up at 560 MHz, 660 MHz, 770 MHz, and 850 MHz.

**Figure 10:**
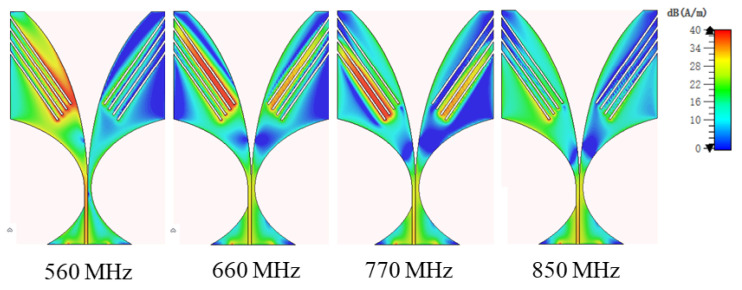
Antenna surface current at different frequencies.

It was found that the four-slot-loaded antenna has the lowest working frequency and widest bandwidth compared to the other scenarios. The surface current of the four-slot-loaded antenna at different resonance frequencies is shown in Figure 10 (560 MHz, 660 MHz, 770 MHz, and 850 MHz). It can be seen from the figure that the current amplitude around the slots $L_{s1}$, $L_{s2}$, $L_{s3}$ is higher at 560 MHz, 660 MHz and 770 MHz, respectively. This clearly indicates that different slots dominate different resonances.

### 4.6. Antenna Realization and Simulation in the Presence of a Layered Phantom

**Figure 11:**
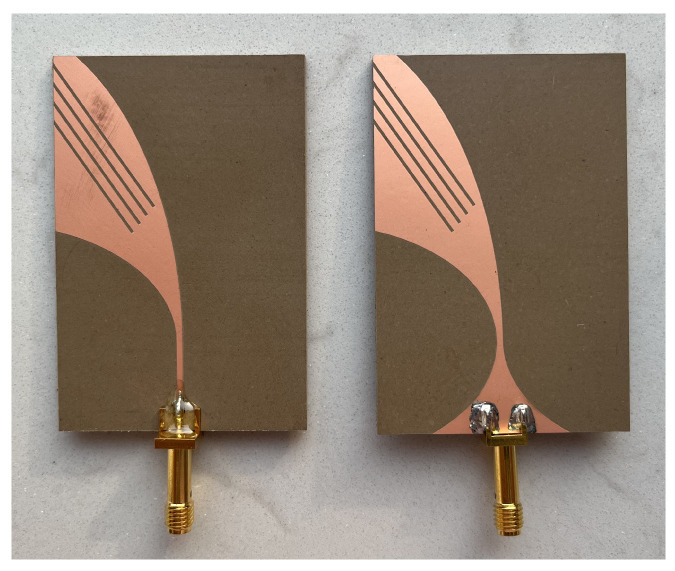
Fabricated antenna.

**Figure 12:**
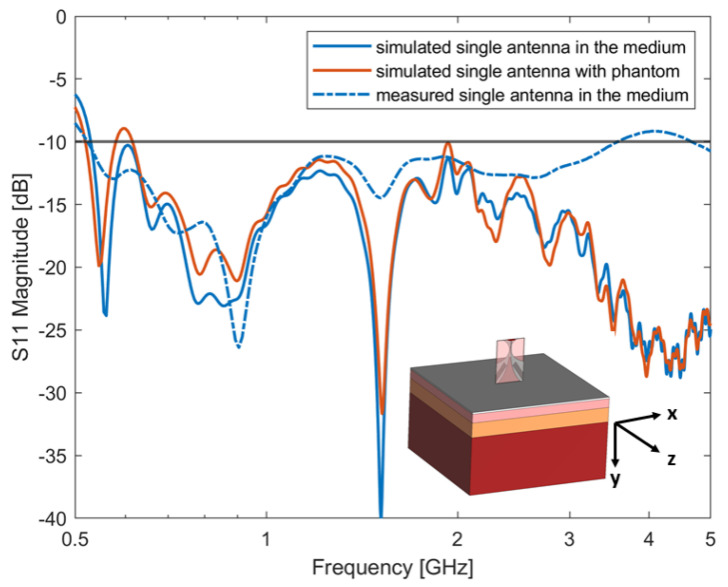
Simulated and measured S-parameters of single antenna in the medium and simulated S-parameters of single antenna with presence of a phantom.

Following the previous analysis, the best-performing antenna was fabricated. According to Table 7, the 4-slot-loaded Vivaldi antenna is 40 mm × 60 mm wide. The antenna was realized by using a single-layered substrate TF1020 (TaiZhou WangLing, Taizhou, China. Thickness $t_{s}$ = 1.905 mm, $\epsilon_{r}$ = 10.2) (see Figure 11). Figure 12 shows the comparison between the simulated and measured $S_{11}$ of the 4-slot Vivaldi antenna. From the figure, an optimum agreement can be drawn up to about 2.5 GHz. It is worth mentioning that the mismatch of the simulation and measurement results above 3 GHz could be due to fabrication errors or antenna soldering issues. The comparison between numerical and experimental results on antenna’s matching can be also considered a measure of the antenna efficiency. In fact, simulations were performed considering perfect electric conductors and a substrate material with no losses. Of course, the actual antenna is made with real materials, which show losses that can decrease efficiency. However, the good agreement between numerical and experimental results show that the main losses are those due to antenna mismatching. Since the antenna design was considered for biomedical microwave imaging applications, to validate the antenna’s performances, the proposed antenna was simulated in front of a layered abdomen phantom (see inset in Figure 12). The phantom consists of four different types of tissues: skin, fat, muscle, and liver. The thickness of each layer is 2.3 mm, 12.2 mm, 20.2 mm and 80 mm, respectively. The thickness of each layer was chosen based on average values [24]. The length and width of the phantom is 200 mm × 200 mm × 114.7 mm, which is sufficiently large to represent typical scenarios in biomedical imaging. The simulated S-parameters of the antenna with and without the presence of the phantom are reported in Figure 12. Figure 12 shows that the antenna’s performance is slightly downgraded with the presence of the layered phantom. However, the antenna bandwidth and impedance are similar.

**Figure 13:**
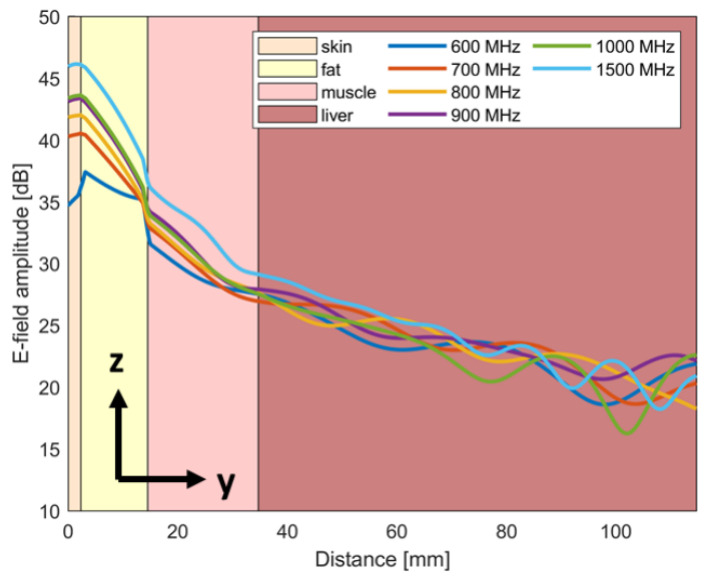
E-field behaviour in the layered phantom along a line in front of the antenna.

The E-field inside the layered phantom at different frequencies is reported in Figure 13. The figure shows the E-field along the *y*-axis (x = 0, z = 0) in front of the antenna.

**Figure 14:**
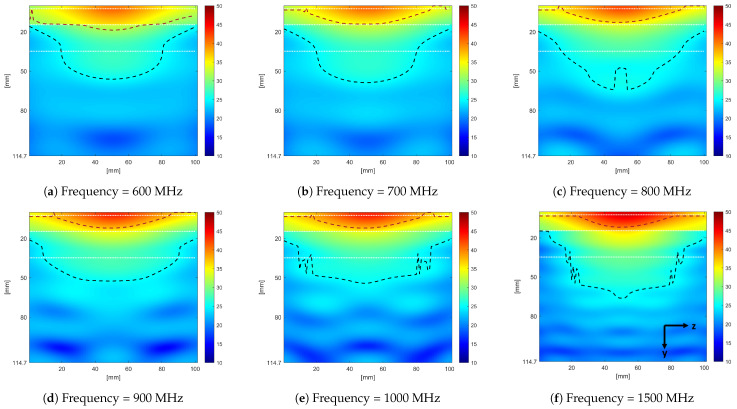
E-field amplitude distribution inside the layered phantom at different frequencies.

The E-field distribution inside the phantom at different frequencies along the y-z plane is reported in Figure 14. In the figure, only the central portion of the phantom, 100 mm wide, is reported for better clarity of the field distribution. In the figure, the white dashed line indicates the boundary between skin, fat, muscle, and liver tissue. The red dashed line indicates an E-field amplitude 6 dB below the maximum value. The black dashed line indicates where the E-field equals 25 dB below the maximum value. It can be seen from the figure that the −25 dB E-field amplitude could penetrate 50 mm beyond the skin layer at different frequencies. The penetration width of the −6 dB E-field amplitude decreases as frequency increases.

### 4.7. Antenna’s Mutual Coupling Study

**Figure 15:**
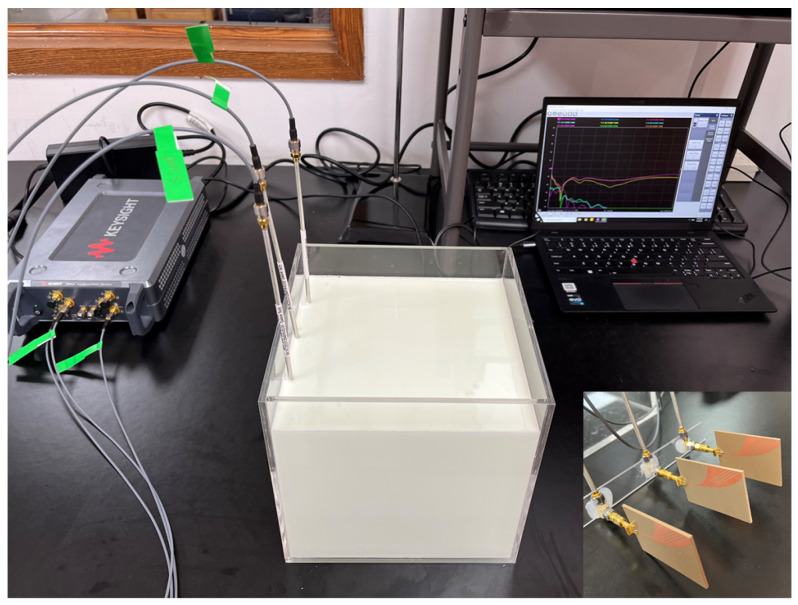
Three-antenna array measurement set-up.

**Figure 16:**
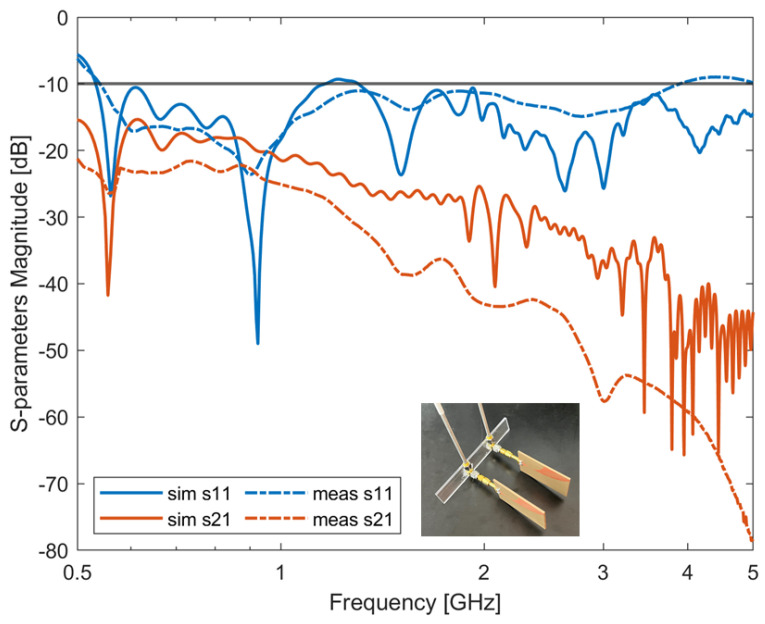
Simulated and measured S-parameters of 2 four-slot-loaded antipodal Vivaldi antenna in array configuration.

**Figure 17:**
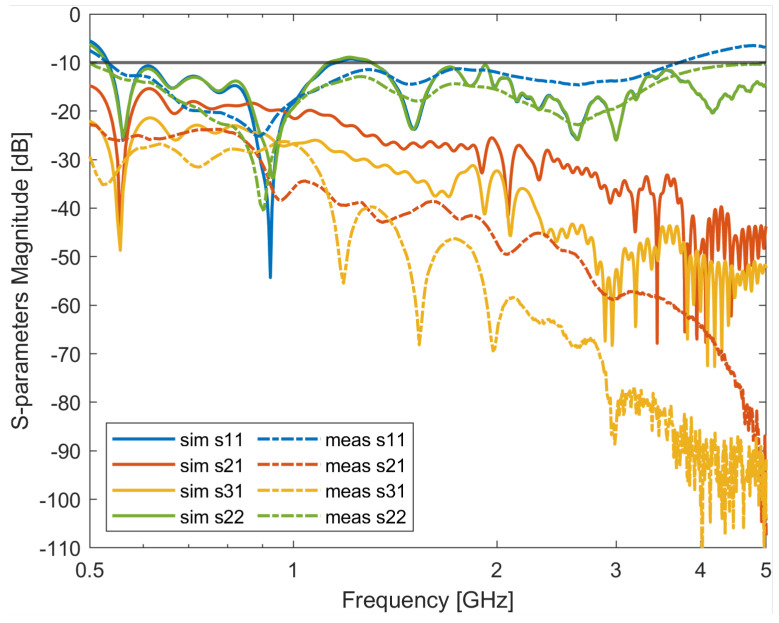
Simulated and measured S-parameters of 3 four-slot-loaded antipodal Vivaldi antenna in array configuration.

To verify the antenna’s mutual coupling when placed in an array, the antennas were simulated in two configurations: two antennas placed in a line (the distance between each element was 50 mm) and three antennas placed in a line (the distance between each element was 50 mm along the *z*-axis—see reference system in Figure 12). The same configurations were also measured with the experimental set-up shown in Figure 15. Figure 16 shows the two-antenna configuration results while Figure 17 shows the three-antenna configuration. Due to the reciprocity and symmetry of the configuration, only the simulated and measured $S_{11}$ and $S_{21}$ parameters of the two-antenna-array scenario are reported in Figure 16. It can be seen from the figure that the antenna’s simulation and measurement results agree with each other, and that the proposed antenna maintains a good performance against mutual coupling when placed in an array configuration (below −15 dB in both cases).

Similarly, from Figure 17, it can be seen that the antenna’s simulation and measurement results agree with each other, and that the proposed antenna maintains a good performance against mutual coupling also when placed in a three-antenna-element array configuration (below—15 dB for both cases). It is worth noticing that the lower experimental Sij (ij = 21 in Figure 16, and ij = 21 and 31 in Figure 17) values can be attributed to higher losses of the experimental set-up with respect to simulations.

The performances and dimensions of the antenna, compared to other Vivaldi antennas from the literature, are reported in Table 8. It is found that the Vivaldi antenna in this work exhibits a wide bandwidth and compact dimensions compared to those in the literature. It is worth mentioning that the antenna was designed for biomedical applications and is intended to operate in the near field within a coupling medium. Therefore, aspects of the antenna’s far-field performance, such as gain and radiation pattern, are not discussed in this work.

## 5. Conclusions

This paper presents the design steps for a slot-loaded Vivaldi antenna tailored for biomedical microwave imaging applications.

While Vivaldi antennas and the use of slots to reduce their dimensions are well known in the literature, the novelty of this study lies in the analysis of the influence of the different slot parameters (location, inclination angle, length, and number) on the achieved performances. Additionally, a physical interpretation of the achieved results is provided. This systematic approach and detailed analysis of different slot configurations to optimize antenna performances offer initial guidelines for an optimal location of the slots on the antenna radiator that can be applied for designs conceived for different applications. Indeed, several parameters should be optimized so that there is a great chance to obtain sub-optimal designs from software optimizations. Furthermore, the investigation combines several strategies, besides the slots, including the use of a high-permittivity substrate material with $\epsilon_{r}=10.2$, and placing the antenna within a coupling medium with $\epsilon_{r}=23$. High-permittivity materials shorten the electromagnetic wavelength, enabling the antenna to operate at the desired frequency range with a smaller physical footprint. Additionally, the coupling liquid allows matching with the biological tissue, reducing electromagnetic field reflections. With a focus on the lowest working frequency and widest bandwidth, the optimal design is identified.

In the final phase, the antenna is fabricated and characterized for performance within the coupling medium. The results from both simulation and measurement exhibit agreement affirming the effectiveness and reliability of the proposed design. Notably, measurements demonstrate that the slot-loaded design substantially mitigated mutual coupling effects when the antenna was placed in an array configuration, enhancing the antenna’s suitability for its intended imaging application. Additionally, the final prototype shows the minimum dimensions with respect to other designs proposed in the literature (Table 8).

To conclude, this work contributes insights into the development of slot-loaded Vivaldi antennas for biomedical microwave imaging, offering a promising solution for enhanced imaging capabilities in the relevant frequency range. The main limitation of the proposed antenna is that it must operate within a coupling medium, which reduces its suitability for wearability in biomedical applications. Future works include realizing the experimental set-up of an imaging system with the proposed antenna.

## Figures and Tables

**Table 1:** Basic antipodal Vivaldi antenna element design parameters.

Parameters	Values (mm)	Parameters	Values (mm)
$W_{a}$	40	$L_{a}$	60
$W_{p}$	32.9	$L_{m}$	14.5
$W_{g}$	21.7	$R_{1}$	19.1
$W_{m}$	0.9	$R_{2}$	17.7
$D_{s}$	27.7	$t_{s}$	1.905

**Table 2:** Single-Slot-Loaded Antipodal Vivaldi Antenna Design Parameters Concerning Different Inclination Angles θ.

Parameters	Values
$D_{1}$ [mm]	8
$L_{s}$ [mm]	25
$W_{s}$ [mm]	0.5
θ [degree]	25°, 30°, 35°, 40°

**Table 3:** Single-slot-loaded antipodal Vivaldi antenna design parameters concerning different slot lengths $L_{s}$.

Parameters	Values
$D_{1}$ [mm]	8
$L_{s}$ [mm]	23, 25, 27, 29
$W_{s}$ [mm]	0.5
θ [degree]	35°

**Table 4:** Single-slot-loaded antipodal Vivaldi antenna design parameters concerning different slot height $D_{1}$.

Parameters	Values
$D_{1}$ [mm]	1, 3, 5, 8, 10, 12
$L_{s}$ [mm]	27
$W_{s}$ [mm]	0.5
θ [degree]	35°

**Table 5:** Two-slot-loaded antipodal Vivaldi antenna design parameters with respect to different slot lengths $L_{s1}$ and $L_{s2}$.

Parameters	No Slot	One Slot (Ref)	Two Slots, $L_{s1}=L_{s2}$	Two Slots, $L_{s1}<L_{s2}$	Two Slots, $L_{s1}>L_{s2}$ (Opt)
$D_{1}$ [mm]	N/A	3	1	1	1
$D_{2}$ [mm]	N/A	N/A	2	2	8
$L_{s1}$ [mm]	N/A	27	27	26	28
$L_{s2}$ [mm]	N/A	N/A	27	27	25
Bandwidth	620 MHz–5 GHz	560 MHz–5 GHz	555 MHz–5 GHz	550 MHz–5 GHz	540 MHz–5 GHz

**Table 6:** Three-slot-loaded antipodal Vivaldi antenna design parameters with respect to different slot lengths $L_{s1}$, $L_{s2}$, and $L_{s3}$.

Parameters	Two Slots	Three Slots	Three Slots	Three Slots	Three Slots	Three Slots
	$L_{s1}>L_{s2}$ (Ref)	$L_{s1}=L_{s2}=L_{s3}$	$L_{s1}>L_{s2}>L_{s3}$ (Opt)	$L_{s1}>L_{s2}=L_{s3}$	$L_{s1}=L_{s2}>L_{s3}$	$L_{s1}=L_{s3}>L_{s2}$
$D_{1}$ [mm]	1	1	1	1	1	1
$D_{2}$ [mm]	8	3	3	3	3	2
$L_{s1}$ [mm]	28	24	28	27	27	26
$L_{s2}$ [mm]	25	24	26	24	27	24
$L_{s3}$ [mm]	N/A	24	24	24	23	26
Bandwidth	540 MHz–5 GHz	560 MHz–5 GHz	535 MHz–5 GHz	540 MHz–5 GHz	540 MHz–5 GHz	550 MHz–5 GHz

**Table 7:** Four-slot-loaded antipodal Vivaldi antenna design parameters.

Parameters	Values (mm)	Parameters	Values (mm)
$W_{a}$	40	$L_{a}$	60
$W_{p}$	32.9	$L_{s1}$	28
$W_{g}$	21.7	$L_{s2}$	26
$W_{m}$	0.9	$L_{s3}$	24
$W_{s}$	0.5	$L_{s4}$	22
$D_{1}$	1	$R_{1}$	19.1
$D_{2}$	2	$R_{2}$	17.7
$D_{s}$	27.7	$t_{s}$	1.905
$L_{m}$	14.5	θ	35°

**Table 8:** Comparison of antenna performance with previous designs.

Ref.	Working	Relative	Efficiency	Dimension	Substrate	Use of	Number
	Frequency	BW (%)		( λ×λ)	Material	Coupling	of Slots
	(GHz)					Medium	
[17]	2.3–18	154	N/A	0.37 × 0.5	FR4	No	10
[27]	3.1–10.6	121	N/A	0.65 × 1.17	RT 6010	Yes	10
[28]	2.4–14	141	N/A	0.38 × 0.48	FR4	No	2
[29]	2.8–6	72.7	N/A	0.37 × 0.42	RO 3010	No	2
[30]	1.6–4	114	N/A	0.35 × 0.44	RT 3010	No	13
[31]	0.5–6	164	N/A	0.54 × 0.84	RO 3010	Yes	11
[32]	3–12	120	>80%	0.32 × 0.35	FR4	No	3 circular slot
[33]	1.7–9	149	N/A	0.23 × 0.24	FR4	No	EBG circular slot
This work	0.53–5	163	N/A	0.34 × 0.51	TF1020	Yes	4

## Data Availability

Data are contained within the article.
